# Sodium salt-assisted low temperature activation of bentonite for the adsorptive removal of methylene blue

**DOI:** 10.1038/s41598-022-06254-z

**Published:** 2022-02-15

**Authors:** Siti Fairos Ab Shattar, Keng Yuen Foo

**Affiliations:** River Engineering and Urban Drainage Research Centre (REDAC), Engineering Campus, Universiti Sains Malaysia, 14300 Nibong Tebal, Penang Malaysia

**Keywords:** Chemistry, Engineering

## Abstract

The sodium salt-assisted low temperature activation of bentonite (BB) was attempted. The unique features of the raw bentonite and BB were characterized with respect to the morphological, functional, and textural analysis. The adsorptive behaviour was evaluated by adopting methylene blue (MB) as the model pollutant via batch adsorption experiment. The experimental data were fitted to the non-linear isotherm equations (Freundlich, Langmuir, and Temkin), while the adsorption modelling was interpreted by the pseudo-first order, pseudo-second order and Elovich models. The adsorptive mechanism was ascertained according to intraparticle-diffusion and boyd models. The intercalation of sodium salt into the bentonite surface give rise to the specific surface area and total pore volume from 120.34 to 426.91, m^2^/g and 0.155 to 0.225 cm^3^/g, respectively, indicating a large proportion of the newly formed surfaces may be connected to new pore walls, associated with the silanol (≡SiOH), and aluminol (≡AlOH), and hydroxyl (–OH) groups for the possible entrapment MB onto the adsorbent. The equilibrium data was satisfactory described by the Langmuir isotherm and pseudo-second order model, with a monolayer adsorption capacity for MB of 318.38 mg/g, while the thermodynamic study verified spontaneous, feasible, and endothermic of the adsorption process.

## Introduction

In general, bentonite, a multi-components mixture of clay and minerals of aluminium phyllosilicate (hydrated aluminasilicate), is a two-dimensional nano-material, which fall under the smectite-class mineral of the montmorillonite^1^. Consist of a multi-expandable layered structure, bentonite owns a net negative surface charge, mainly ascribed to the isomorphous substitution of Al^3+^ for Si^4+^, and Mg^2+^ for Al^3+^ within the tetrahedral and octahedral clay framework. The charge could be compensated by the exchangeable cations (Na^+^ and Ca^2+^), which are known to be the inter-layer hydrated cations^2^. The unique feature of bentonite is the formation of thixotrophic gels in the aqueous medium to enlarge its volume at 12–15 times of its dry bulk. When bentonite is in contact with water, the layered structure will expand with the hydration of Na^+^ and Ca^2+^, to turn into a hydrophilic characteristic^3^. The adsorptive behavior of the natural bentonite could be specifically altered for the targeted pollutants. One of the possible route is the exchange of quaternary ions (R_4_N)^+^ with the Group IA and IIA metal ions present between the aluminum and silica layers^4^. The reduction of the hydrophilicity could be represented by the expulsion of metal ions with the coordinated water molecules, and in the alkaline conditions, this negative load would be screened, which in turn favouring the approximation of the tactoids, and rearrangement of the clay particles^5^. Therefore, different routes have recently been proposed by the environmentalists to enhance the adsorption capability of the bentonite derivatives.

Amongst methods of beneficiation for bentonite adsorption process are pillaring, calcinations, acid treatment and salt exchange. These modifications may involve the changing of textural or structural properties, specific surface area, and porosity, following the incorporation of new functionalities or compositional changes to generate and/or strengthen the surface-active sites. The intercalation of hydroxyl aluminum, iron, titanium, chromium and zirconium cations, also known as basal plane spacing (001) or pillaring step may expand the clay layer, to be transformed into stable porous adsorbents. However, the pillaring solutions usually takes a longer preparation duration of 3 h to 10 day^6^. Calcination could assist in the thermal treatment for better adsorptive uptake of clay minerals, driven by the loss of adsorbed moisture, structural changes, textural properties, and dispensability of bentonite in the aqueous solution^7^. Nevertheless, the applied treatment process and dehydration reaction with the collapse of the clay structure may remove the non-clay matter, subsequent by the octahedral cation movement within the interlayer clay sheets. Acid treatment involves the penetration of protons into the interlayer of bentonite substituting the exchangeable cations of the clay minerals, and improve the porosity, surface active sites and mineral purity of the external surface^8^. The partial dissolution effect of the excessive acid treatment however, may reduce the adsorptive uptake by at least 30%. Therefore, a call for the continuous research in establishing a simple modification technique, eco-friendly, with less energy consumption, time saving, and effective for environmental conservation is deeply required.

In a study conducted by Kaufhold and Dohrmann^9^, a close reaction system between 36 bentonites and NaCl solution (6 M) was conducted at 60 °C for 5 months. Compositional analysis indicated that no obvious mineral alteration was detected, while the bentonite derivatives were highly stable in the NaCl solutions to support the reaction with exceeding 24 weeks. The reported results were supported by Stawiński et al.^10^ that no irreversible changes could be identified of the sodium salt treated adsorbent, indicating redeposition of the dissolute elements as hydroxides. These Al, Mg and Fe hydroxides that could be adsorbed by the broken edge sites, or present concurrently may appear as the interlayer or coating surface between the clay minerals. The reactivity of this phase is significantly higher than the free hydroxides, contributed by the three major surface binding sites, notably permanent charged siloxane surface, mono-coordinated and newly formed hydroxide species within the clay edges^11^. With the aforementioned, in this work, the sodium salt-assisted low temperature activation of highly porous bentonite derived adsorbent (BB) was carried out by varying the modification ratio. The specific features were evaluated with respect to the morphological characteristic, surface functionalities and textural property. Methylene blue was adopted as a model adsorptive behaviour. Non-linear isotherm models and kinetic equation were applied for the data simulation. The adsorptive mechanism was ascertained by the intra-particle diffusion and Boyd models, whereas the thermodynamic behavior were critically elucidated.

## Methodologies and materials

### Functionalized bentonite

Raw bentonite applied in this work was commercially acquired from Sigma Aldrich, Malaysia. The activation step was performed by mixing the raw bentonite with NaCl solution at 70 ℃ for 1 h, with the impregnation ratio (IR) given by:

1 $${\text{IR = }}\frac{{W_{{{\text{Bentonite}}}} }}{{W_{{{\text{NaCl}}}} }}$$

where *W*_bentonite_ and *W*_NaCl_ are the dry mass of bentonite (g) and NaCl (g). The activated bentonite (BB) was collected by centrifugation, subsequent by washing extensively with deionized water (DW) until the washing solution reached to a neutral pH before it was dried and stored in a sealed bottle.

### Model pollutant

Methylene blue (MB), a model pollutant with low biodegradability in nature was adopted. The chemical structure, molecular weight and *λ*_max_ of MB are derived as C_16_H_18_C·N_3_S_3_.H_2_O, 373.91 g/mole and 663 nm, respectively. It could be dissociated into a MB^+^ cation and a Cl^−^ anion in the aqueous medium, with the dehydrated features of 14.3 Å in width, 6.1 Å in depth, 4 Å in thickness, and the molecular volume and molecular diameter of 241.9 cm^3^/mole and 0.8 nm, respectively. An appropriate amount of MB was dissolved in DW for preparation of standard stock solution, and working solutions was acquired by successive dilutions.

### Adsorption experiments

The adsorption experiments were performed in the conical flasks containing 200 mL of adsorbate solution and 0.2 g of BB at the pre-determined concentrations from 50 to 500 mg/L. The solution mixture were agitated at 130 rpm and 30 °C for 24 h, and the concentration of the supernatant solution was analyzed at 663 nm using a UV–Vis spectrophotometer (Shimadzu-1800). The experiments were conducted in triplicates, and the equilibrium uptake, *q*_e_ (mg/g), was determined by:

2 $$q_{{\text{e}}}^{{}} = \frac{{(C_{{0}}^{{}} - C_{{\text{e}}}^{{}} )V}}{W}$$

where *C*_0_, and *C*_e_, (mg/L) are the initial and equilibrium liquid-phase concentration of MB, and *V* and *W* is denoted to volume of adsorbate solution (L), and dry mass of BB (g), respectively.

To better correlate the experimental data with the isotherm equations, the non-linearized Langmuir^12^, Freundlich^13^ and Temkin^14^ isotherm models have been adopted:

3 $$q_{{\text{e}}}^{{}} = \frac{{Q_{0}^{{}} K_{{\text{L}}}^{{}} C_{{\text{e}}}^{{}} }}{{1 + K_{{\text{L}}}^{{}} C_{{\text{e}}}^{{}} }}$$

4 $$q_{{\text{e}}}^{{}} = K_{{\text{F}}}^{{}} C_{{\text{e}}}^{1/n}$$

5 $$q_{{\text{e}}}^{{}} = B\ln (AC_{{\text{e}}}^{{}} )$$

where *Q*_0_ (mg/g), *K*_L_ (L/g) and *K*_F_ (mg/g) (L/mg)^1/*n*^ are the Langmuir isotherm constants for adsorption capacity, adsorption energy, and Freundlich isotherm constant respectively, where 1/*n* is a measure of adsorption intensity, and *B* = *RT*/*b*_T_, with *b*_T_ (J/mole), *R* (8.314 J/mole *K*), *T* (K) and *A* (L/mole) are the heat of sorption, universal gas constant, absolute temperature and equilibrium binding constants, respectively.

For the assessment of the non-linear isotherm models, a trial-and-error procedure was performed to maximize the coefficient of determination *R*^2^, between the experimental data and isotherms in the solver add-in with Microsoft’s spreadsheet, Microsoft Excel, defined as:

6 $$R^{2} = \frac{{(q_{{\text{e,meas}}} - \overline{q}_{{\text{e,calc}}} )^{2} }}{{\sum {(q_{{\text{e,meas}}} - \overline{q}_{{\text{e,calc}}} )^{2} + (q_{{\text{e,meas}}} - q_{{\text{e,calc}}} )^{2} } }}$$

where *q*_e,calc_, $\overline{q}$_e,calc_ and *q*_e,meas_ are the predicted, average mean and measured methylene blue concentration at equilibrium deduced from the isotherm model. The experimental data then was validated by the root-mean-square deviation (*RMSD*), given by:

7 $$RMSD = \frac{{\sqrt {\sum\limits_{i = 1}^{n} {(q_{{{\text{exp}}}}^{{}} - q_{{\text{p}}}^{{}} )^{2}} }_{{}} }}{n - 1}$$

where *n* indicating the total data points, while the experimental and predicted adsorption capacity are represented by corresponding *q*_exp_ (mg/g) and *q*_p_ (mg/g), respectively.

The changing solution pH on the adsorptive performance was conducted at the solution pH from 1 to 12, at the BB dosage of 0.2/200 mL, initial concentration at 500 mg/L and operating temperature of 30 °C. The pH adjustment was conducted by using 0.1 M hydrochloric acid (HCl) and/or sodium hydroxide (NaOH), and the measurement was ascertained using a pH meter.

### Kinetic modelling

For the interpretation of kinetic analysis, the solution samples were measured at prescribed time interval, and the adsorptive uptake at time *t*, *q*_t_ (mg/g), was determined by:

8 $$q_{{\text{t}}}^{{}} = \frac{{(C_{0}^{{}} - C_{{\text{t}}}^{{}} )V}}{W}$$

where *C*_t_ (mg/L) is denoted as the liquid-phase concentration of MB dye at time, *t*.

It describes the adsorbate uptake, mass transfer, and the equilibrium time of an adsorption process. In this study, the pseudo-first order^15^, pseudo-second order^16^ and Elovich kinetic^17^ equations were adopted for the effective simulation of the equilibrium data, defined as:

9 $$\ln (q_{{\text{e}}}^{{}} - q_{{\text{t}}}^{{}} ) = \ln q_{{\text{e}}}^{{}} - k_{1}^{{}} t$$

10 $$\frac{t}{{q_{{\text{t}}}^{{}} }} = \frac{1}{{k_{{2}}^{{}} q_{{\text{e}}}^{{2}} }} + \frac{1}{{q_{{\text{e}}}^{{}} }}t$$

11 $$q_{{\text{t}}}^{{}} = (\frac{1}{b})\ln (ab) + \frac{1}{b}\ln t$$

where *k*_1_ (1/h) and *k*_2_ (g/mg h) is the adsorption rate constant for the pseudo-first and pseudo-second order kinetic equation respectively, while *a* (mg/g h) is the initial sorption rate and *b* (g/mg) is related to the extent of surface coverage, and activation energy for chemisorption of the Elovich equation. The suitability of the kinetic models was validated by the correlation coefficient, *R*^2^, and the normalized standard deviation, Δ*q* (%):

12 $$\Delta q(\% ) = \sqrt {\frac{{\sum {\left| {(q_{{\text{e,exp}}}^{{}} - q_{{\text{e,cal}}}^{{}} )/q_{{\text{e,exp}}}^{{}} } \right|_{{}}^{2} } }}{n - 1}} \times 100$$

where the data points, the experimental and calculated adsorption capacity are represented by *n*, *q*_e,exp_ (mg/g) and *q*_e,cal_ (mg/g), respectively.

### Adsorption mechanism

The kinetic study explains solute-adsorbent surface interaction during the adsorption process, but does not elucidate the solutes particles diffusion mechanism onto the adsorbent surface. Therefore, the equilibrium data was analyzed according to the Fick’s second diffusion law, the Weber and Morris intra-particle diffusion^18^ and the Boyd film diffusion models^19^:

13 $$q_{{\text{t}}}^{{}} = k_{{{\text{pi}}}}^{{}} t_{{}}^{0.5} + C_{{\text{i}}}^{{}}$$

14 $$B_{{\text{t}}}^{{}} = - 0.4977 - \ln (1 - F)$$

where the diffusion rate constant and thickness of the boundary layer could be devoted as *k*_pi_ (mg/g h^0.5^) and *C*_i_, respectively, whereas *B*_t_ is a mathematical function of fractional attainment of equilibrium *F*, at any time *t*, given by:

15 $$F = \frac{{q_{{\text{t}}}^{{}} }}{{q_{{\text{e}}}^{{}} }}$$

### Thermodynamic study

Thermodynamic considerations are required for determination of spontaneity nature, high feasibility or energy requirement of an adsorption process^20^. The Gibbs free energy change, Δ*G*° (kJ/mole) represents the spontaneity assessment of a chemical reaction, and both changing entropy and energy must be critically identified. Accordingly, the negative Δ*G*° indicates a high spontaneity and energy of a reaction, specific at a temperature, and the adsorption equilibrium constant *K*_L_ could be derived by:

16 $$\Delta G^\circ = - RT\ln K_{{\text{L}}}^{{}}$$

with *R* (8.314 J/mole *K*) is the universal gas constant, and *T* (*K*) is the absolute temperature. The relation of the equilibrium constant with the changing operating temperature could be acquired from the differential equation proposed by Ho et al.^21^:

17 $$\frac{{D\ln K_{{\text{L}}}^{{}} }}{{{\text{d}}T}} = \frac{\Delta H^\circ }{{RT_{{}}^{2} }}$$

The integrated Eq. (17) yields:

18 $$\ln K_{{\text{L}}}^{{}} = - \frac{\Delta H^\circ }{{RT}} + Y$$

where *Y* is a constant, and Eq. (18) could be further rearranged in the form of

19 $$- RT\ln K_{{\text{L}}}^{{}} = \Delta H^\circ - TRY$$

Let $$\Delta S^\circ = RY.$$

Substituting Eq. (16) into Eq. (19), Δ*G*° can be represented by:

20 $$\Delta G^\circ = \Delta H^\circ - T\Delta S^\circ$$

where Δ*H*° and Δ*S*° are the standard enthalpy and standard entropy change, respectively.

### Physio-chemical characterization

The morphological structure of the functionalized adsorbent was examined by using the scanning electron microscope (Supra 35 VP, Germany) equipped with W-Tungsten filament, with MnK_α_ as the energy source. The porosity measurement was conducted by the nitrogen adsorption isotherm using the Micromeritics ASAP 2020 analyzer. The Fourier transform infrared spectroscopy (FTIR) analysis was conducted according to the KBr method using the Perkin-Elmer Spectrum GX infrared spectrometer in the scanning range of 4000–400 cm^−1^. The point of zero charge, *pH*_pzc_ was justified by adjusting the solution pH of 200 mL of 0.01 M NaCl solution to a value between 1 and 12. The final pH was measured after 2 days agitation. The point where *pH*_initial_  − *pH*_final_ = 0 is *pH*_pzc_.

## Results and discussions

### Chemical impregnation ratio, IR

**Figure 1:**
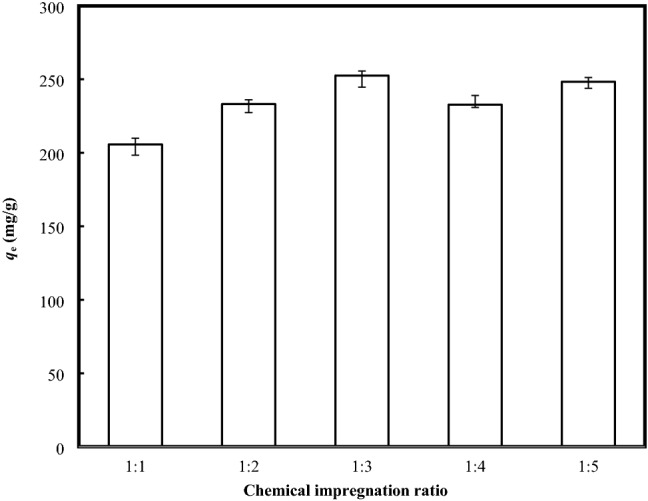
Effects of chemical impregnation ratio on the adsorptive uptake of MB (Adsorbent dosage = 0.2 g/200 mL; Temperature = 30 °C; Initial concentration = 500 mg/L).

A feasible approach to enhance the adsorptive performance of the clay mineral is the alteration of surface chemistry, in which activation agents play a decisive role. During the activation process, the pore system of the clay minerals could be modified, leading to the changing catalytic, adsorptive and environmental functions of the minerals with the improvement on the crystalline structure of the minerals via structural ion dissolution or reorganization of the interior porosity. The effect of *IR* for the equilibrium uptake of MB onto BB is depicted in Fig. 1. It can be clearly found from Fig. 1 that increasing the IR from 1:1 to 1:3 indicated a steadily rise of the adsorption uptake from 205.67 to 252.59 mg/g, with the best IR recorded at 1:3. The phenomena may be ascribed to the rising ionic force with the incorporation of sodium salt into the clay structure, that has induced a double layer compression to assist the process of approximation and association of the clay structure. Therefore, the dye molecules which were initially bonded as the aggregates or monomers onto the external surface of the clay minerals during the association process, could be re-located into the internal region. Cione et al.^5^ further stated that, the electrostatic repulsion between the negative charged of the clay particles would be screened in the presence of salt, that in turn reduced the repulsive interaction, favouring the approximation between the tactoids, resulting in the entrapment of MB as the monomers or aggregates molecules.

In contrast, the subsequent increase in the IR ratio, beyond the optimum value showed a gradually decrease in the adsorptive uptake of MB. The excessive presence of sodium salt would promote vigorous reaction within the clay particles, which destroyed the clay framework, resulting in a dramatic reduction of the accessible surface binding sites. The adsorption mechanism, which has been reported to occur partly by ion exchange releasing cations in the interlayer and basal plane surfaces, and partially via non-columbic interaction between the bentonite surface neutralized site and the adsorbed cation, is greatly governed by the changing surface polarity, compression layer, or ionic strength of the negatively charged adsorbent. In this sense, the excessive deposition of sodium would introduce a repulsive force to the cationic MB molecules, resulting in the reduction of the adsorptive uptake.

### Effect of initial concentration and contact time

**Figure 2:**
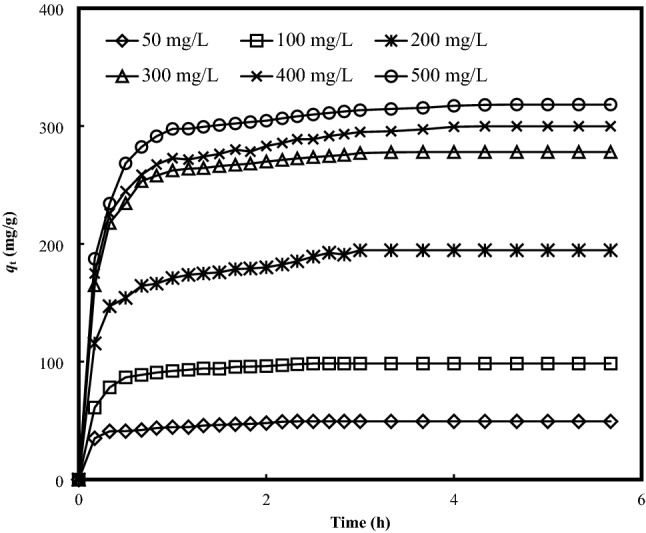
Effect of initial concentrations and contact time on the adsorptive uptake of MB onto BB (Adsorbent dosage = 0.2 g/200 mL; Temperature = 30 °C; Initial concentration = 50–500 mg/L).

The effect of initial concentration and contact time for the adsorptive uptake of MB onto BB at 30 °C is displayed in Fig. 2. It could be observed that the adsorption process increased rapidly at the initial time intervals, before the transitional phase took place, and reached to a plateau. At the initial stage, the adsorption uptake rate increased significantly, signalling the presence of readily accessible surface binding sites^22^. As the equilibrium approached, the process turned slower, where the maximum adsorptive uptake under the operating conditions reflects the equilibrium uptake. From the present result, it can be inferred that the rising adsorptive uptake from 49 to 318 mg/g with the increasing initial MB concentration from 50 to 500 mg/L, may ascribed to a higher concentration gradient to overcome the mass transfer resistance between the aqueous MB solution and the solid BB.

Furthermore, in the MB-BB suspension system, the adsorptive uptake of MB dye molecules to the external surface of BB would result in the rising local concentration of MB dye, with the formation of dye aggregates^23^. These MB molecules would further migrate to the interlamellar region, with the disaggregation of MB aggregates, and restoration of the MB monomers^24^. At the higher loading of MB, these agglomerates of dye molecules are predicted to be dominant, while these monomers or dimers would be practically absent in the MB-BB complexes. Additionally, it can be found that a longer contact time was required for a higher MB concentration to attain the equilibrium. The obtained findings could be depicted by the diffusion of MB molecules across the boundary layer, into the internal structure of adsorbent, and to the binding sites^25^, suggested a smooth and monolayer coverage of MB dye onto BB.

### Effect of solution pH

**Figure 3:**
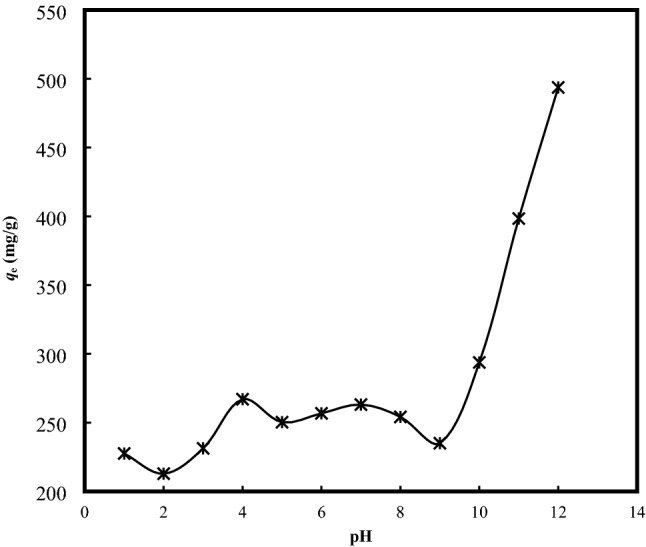
Effect of solution pH on the adsorptive uptake of MB onto BB (Adsorbent dosage =  0.2 g/200 mL; Temperature = 30 °C; Initial concentration = 500 mg/L).

The changing solution pH remains one of the most influencing parameters determining the performance of the adsorption process. The response is particularly due to influence of the hydrogen ions which may affect the surface charges and ionization of the functionalities of the solid adsorbent^26^. Figure 3 shows the changing adsorptive uptake of MB onto BB as a function of solution pH. In the aqueous medium, cationic dye including MB tend to produce the reduced form of CH^+^ and C^+^ cations^27^. The negatively surface charged surface binding sites within the clay based adsorbent would favor the adsorption of the cationic MB dye, driven by the electrostatic attraction force. Lowering the pH range would generate a positive charged or protonated surface on the clay adsorbent, with a dramatic electrostatic repulsion effect hindering the adsorptive interaction, and further amplified by the competing effect between the dye cations and the effect of excess H^+^ ions for the surface binding sites^28^. On the other hand, the higher pH values within the basic medium would induce a negative charge to the surface BB, in strengthening the electrostatic interactions between the cationic MB and the negatively charged BB^29^. With the *pH*_zpc_ of 9, the BB surface turned positive at the pH below 9, and showed a net negative charge at the pH above the *pH*_zpc_, which supported the adsorption of cationic MB onto the negatively charged BB via electrostatic attraction after the modification process as described in Eq. (21).

21 $${\text{BB-SiO}}^{ - } + {\text{MB}}^{ + } \leftrightarrow {\text{BB-SiO}}^{ - } - -^{ + } {\text{MB}}$$

Specifically, the potential surface charge of the clay-aqueous system is mainly governed by the activity of the ions or the newly formed ion complex with H^+^ and OH^−^ derived as:

22 $${\text{SiOH}} + {\text{OH}}^{ - } \to {\text{SiOH}}^{ - } + {\text{H}}_{{2}}^{{}} {\text{O}}$$

In this study, the maximum adsorptive uptake of MB onto BB was found to be at the basic medium, where an extremely high cation exchange capacity was recorded, and driven by the basal oxygen surface of the tetrahedral sheets with the MB solution, which contain excess hydroxyl ion. The highly saline solution at the alkaline environment was expected to contribute to the protonation/deprotonation of the surface hydroxy site (M-OH), not limited to the alumina group of the BB, which gave rise to the more negative charges.

23 $${\text{H}}_{{2}}^{{}} {\text{O}} + {\text{M-O}}^{ - } \mathop {\underset {{\text{OH}}^{ - } } \longleftrightarrow }\limits^{{{\text{H}}^{ + } }} {\text{M-H}}\mathop {\underset {{\text{OH}}^{ - } } \longleftrightarrow }\limits^{{{\text{H}}^{ + } }} {\text{M-OH}}_{{2}}^{ + }$$

These potential determining ions are represented by H^+^ and OH^-^, and this aforementioned electrostatic force between of the negatively charged BB and the positively charged MB would result in a greater uptake of cationic dyes.

### Isotherm modelling

The modelling is the development of representative equation of the adsorption system, which could be applied for the design purposes^30^. It is well known that, the estimation of isotherm parameters from the non-linear isotherm modelling is preferred in minimizing the data distribution errors between the experimental and predicted parameters or fit distortions of the linearization models^31^. Three non-linear equilibrium isotherms, specifically Langmuir, Freundlich, and Temkin isotherms have been adopted. Each theoretical plots of the modelling have correlated with the experimental data, and further evaluated by the correlation coefficients (*R*^2^) and Root Mean Square Deviation (*RMSD*). The isotherm parameters with the correlation *R*^2^ and *RMSD* are tabulated in Table 1. The presented data was most suitable described by the Langmuir isotherm, with the highest *R*^2^ value of 0.998, and the lowest *RMSD* value of 1.70. The best fit to the Langmuir isotherm model suggested the homogeneous nature, energetically equivalent and identical of the surface binding sites, with the corresponding *Q*_0_ and *K*_L_ of 318.38 mg/g and 0.29 L/mg, respectively.

**Table 1:** Adsorption isotherm parameters for the adsorption of MB onto BB at 30 °C.

Isotherm	Parameter constants	
Langmuir	*Q*_0_ (mg/g)	318.38
	*K*_L_ (L/mg)	0.29
	*R*^2^	0.998
	*RMSD*	1.70
Freundlich	*K*_F_ ((mg/g)(L/mg)^1/n^)	112.43
	*n*	4.65
	*R*^2^	0.827
	*RMSD*	15.53
Temkin	A (L/g)	6.33
	*B*	48.23
	*R*^2^	0.949
	*RMSD*	9.46

The equilibrium data was further analyzed with respect to the separation factor *(R*_L_), a dimensionless constant proposed by:

24 $$R_{{\text{L}}}^{{}} = \frac{1}{{1 + K_{{\text{L}}}^{{}} C_{0}^{{}} }}$$

whereby *R*_L_ is a quantitative verification on the favourability of the adsorption system where *R*_L_ = 0 indicates irreversible, 0 < *R*_L_ < 1 represents favorable, *R*_L_ = 1 is linear and *R*_L_ > 1 corresponds to unfavourable interaction. From Supplemental Fig. S1, it has been found that the calculated *R*_L_ ranged between 0.01 and 0.1 at the initial concentration range of 50–500 mg/L, indicating favourable of the tested adsorption system. Table 2^32–41^ shows a comparative evaluation of the monolayer adsorption capacity for MB onto different functionalized clay based adsorbent. The clay based adsorbent reported in this research showed a relatively greater performance as compared with the reported literature findings.

**Table 2:** Comparative evaluation of monolayer adsorption capacities for MB onto different clay derivatives.

Clay	Activation method	Activating agent	Monolayer adsorption capacity, (mg/g)	References
Bentonite	Low temperature	NaCl	318	Present study
Bentonite	Ion exchange	Hexadecyltrimenthylammonium chloride	1	Anirudhan and Ramachandran^32^
Palygorskite	Ion exchange	NaOH	136	Wang et al.^33^
Bentonite	Crosslinked	Chitosan	97	Bulut and Karaer^34^
Montmorillonite	Pillaring	Iron oxide	69	Cottet et al.^35^
Montmorillonite	Ion exchange	dodecyl sulfobetaine	150	Fan et al.^36^
Bentonite	High pressure	Attapulgite	110	Liu et al.^37^
Rectorite	Ion exchange	Gemini surfactant (organic)	46	Zeng et al.^38^
Bentonite	Heat treatment	H_2_SO_4_	111	Banat et al.^39^
kaolin	Calcination + Ion exchange	NaOH	17	Ghosh and Bhattacharyya^40^
Montmorillonite	Microwave irradiation	Sulfonated chitosan	188	Abdul Mubarak et al.^41^

### Adsorption kinetics and mechanism study

Adsorption kinetic represents a variable tool elucidating the residence time and controlling step of the adsorptive interaction. The equilibrium data was simulated by pseudo-first order, pseudo-second order and Elovich kinetic equations by adopting the linear plots of ln (*q*_e_-*q*_t_) against *t*, *t*/*q*_t_ versus *t*, and *q*_t_ against ln*t*, respectively. The adsorption data given in Supplemental Table S1 showed a good compliance with the pseudo-second order equation, with the highest *R*^2^ and lowest Δ*q* (%) of 0.999 and 1.08–5.32%, respectively. This suggested that chemisorption may be the rate-limiting step, with electrons sharing between MB cations and the hydrophilic site of BB. However, the experimental *q*_e_ results were deviated significantly from the theoretical value with the pseudo-first order and Elovich kinetic models, implying more than one-step may be involved in the adsorption MB molecules onto BB. Similar correlation has been recorded by previous researches for the adsorption of MB dye onto different functionalized adsorbents^23,42^.

The diffusion mechanism was further accessed using the Weber and Morris^18^ intraparticle diffusion model, which describes the time-dependent diffusion of adsorbate components of the adsorption process. The model implies that particle diffusion is the controlling step if the relationship between the adsorbed adsorbate per unit mass of adsorbent (*q*_t_) versus the square root of time (*t*^1/2^) provide a straight line, and passes through the origin^43^. Specifically, external diffusion, surface diffusion or pore diffusion have been identified to be the major steps, driven by the ion exchange, complexation, precipitation or a combination of their interactions^44^.

The instantaneous adsorption of the first 40 min may be corresponded to the mass transfer of adsorbate molecules from the bulk solution onto the adsorbent surface, subsequent by gradual adsorption stage of the external mass transfer resistance, that was mainly intraparticle diffusion controlled. The alteration is related to the coupling between solid and liquid phases, or the initial and boundary of the interactive system^45^. The final equilibrium stage is the last stage, where the adsorption process started to slow down, and reached to a plateau. From the presented findings, the greater adsorption rate was observed at the initial stage, and it reduced gradually as the equilibrium approached. This changing equilibrium behaviour could be resulted from the dimerization of the MB molecules at the higher initial concentration range or ionization of the MB molecules, with one or more reactions took place during the adsorption interaction^28^.

**Figure 4:**
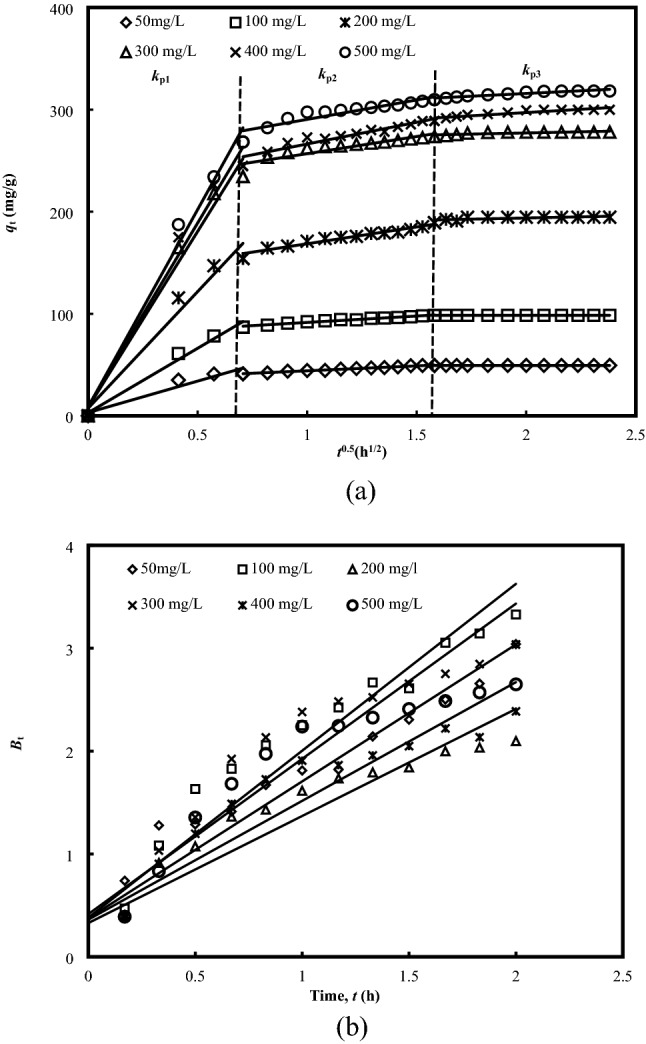
Intraparticle diffusion (**a**) and Boyd plots models (**b**) for the adsorption of MB onto BB at 30 °C.

The results summarized in Supplemental Table S2 provide good agreement that intraparticle diffusion represents the rate controlling step of the adsorption process, supported by the plots *q*_t_ versus *t*^1/2^ plots (Fig. 4a) that moved beyond the origin, and the linear straight lines yields the intercepts *C*_i_. This intercept *C*_i_ reveals the growing thickness of the boundary layer, with larger the *C*_i_ value, indicates the greater effect^46^. If the diffusion could be identified, the boundary layer may be seen as a viscous drag between the adsorbate and BB over the bulk surface, and the higher adsorptive uptakes could be represented by the rising *C*_i_ values.

The experimental data were further fitted to the Boyde’s equation^47^ for predicting the rate controlling phase of the adsorption process. Accordingly, the rate limiting step is film-diffusion or chemical reaction controlled if the plot is linear or non-linear, but do not pass through the origin. Conversely, intraparticle diffusion is the rate controlling step if a straight line passes through the origin is produced. As demonstrated by Fig. 4b, the plots with a linear profile but did not pass through the origin, verified that external transport to be the major rate limiting step, due to the major governance by film diffusion, and external transports at the surface could be more prominent than the internal transport, with the assumption that the controlling mechanism at the surface of BB was a result of the chemical interaction.

### Thermodynamic study

The energy change in term of thermodynamic consideration represents an important marker for the practical application, and provides additional knowledge underlying the inherent energetic different during the adsorptive interaction^48^. The Gibbs free energy change, Δ*G*°, indicates the spontaneity of a reaction and therefore justifies the viability of the adsorptive system. The Δ*G*° for the adsorption system at the tested temperature range were computed by Eq. (20), as depicted in Table 3. Increasing the adsorption temperature from 30 to 50 °C, recorded a steadily decrease of Δ*G*° from − 6.38 to − 9.58 kJ/mol, illustrating greater feasibility of the adsorption at the higher temperature range, indicative of spontaneous nature and low activation energy of the adsorptive interaction. The results were supported by the rising *Q*_0_ from 318.38 to 357.14 mg/g. At the low temperatures region, the competition between the MB and water molecules took place within the interlamellar surface, resulted from the strong hydrophilic feature of the clay layer, and an intensive electrostatically interaction. In this sense, the high charged trimmers (MB^+^)_3_ may involve in ion pairing mechanism for the intensive interaction surface within the active sites of the clay minerals at the low temperature region. The rising temperature however could facilitate the formation of well-defined adsorbed molecules around the exchange sites, driven by delamination of the clay particles, mainly attributed to the greater interactions and changing swelling properties of the bentonite surface.

**Table 3:** Thermodynamic parameters for the adsorption of MB onto BB.

Δ*G*° (kJ/mole)			Δ*H*° (kJ/mole)	Δ*S*° (J/mole *K*)
303 K	313 K	323 K		
− 6.38	− 7.77	− 9.58	42.04	159.55

The physical or chemical interactions depends on the magnitude of Δ*G*°, in which −20 kJ/mole corresponded to the spontaneous physical processes, and the Δ*G*° between − 80 to − 400 kJ/mole is denoted to the chemisorption process^30^. The acquired Δ*G*° for the adsorption system ranging from − 6.38 to − 9.58 kJ/mole, revealed that it is dominated primarily by the physical adsorption mechanism. This similar trend has been supported by Alhumaimess^49^, Oukil et al.^50^, and Uyar et al.^51^ for the adsorption of MB onto vermiculate, modified HUSY zeolite and an amorphous mixture of γ-alumina and silica, and alginate-clay quasi-cryogel beads, respectively. The values of Δ*S*° derived from the plot of Δ*G*° versus *T* was found to be 159.55 J/mole *K*. The positive standard entropy (Δ*S*°) may be related to the hydration of dye cations, reorientation, and restructuring of the clay platelets, and rocketed the high affinity of BB for MB molecules, with structural changes in dyes and BB surface^52^. This positive entropy change was ascribed to the increasing disorder, driven by the delamination of BB, with the formation of an ordered system.

### Morphological structure and surface characteristics

**Figure 5:**
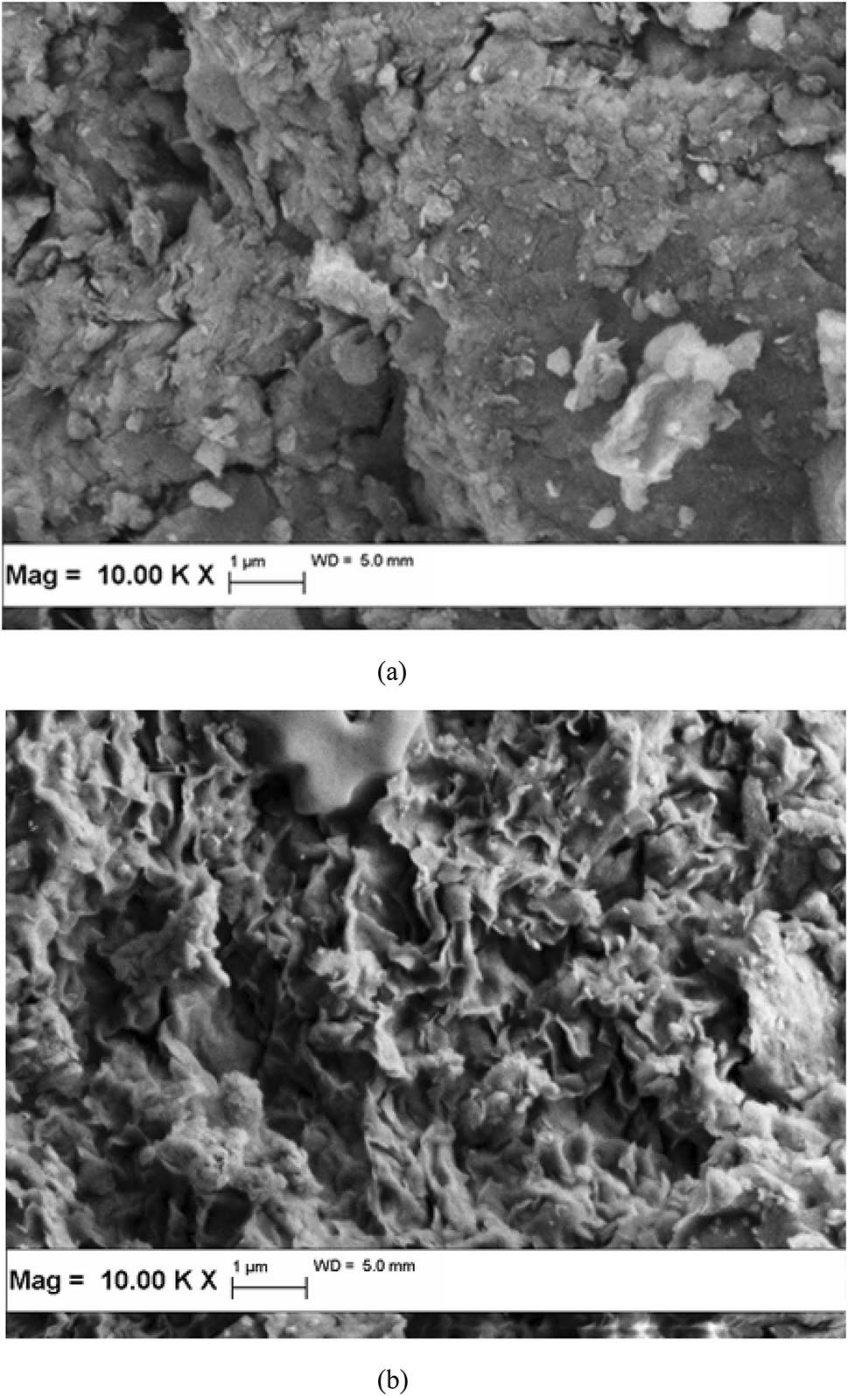
Scanning electron micrographs of (**a**) bentonite and (**b**) BB.

The fundamental physical properties and morphological structure of the functionalized adsorbents was accessed in term of Scanning Electron Microscopy (SEM), as presented in Fig. 5. The smooth appearance of the raw bentonite surface could be closely related to the packed flakes structures. In contrast, BB demonstrated a well developed meso and microstructural porosity. The incorporation of accumulated sodium salt crystals implied a detrimental effect on bentonite structure, to generate a siliceous skeleton with wide quantity of open-air voids, characterized by a broad range of mutual bonds and interfacial zones. The smectite leaflets have been completely de-structured after the chemical modification, with the collapse of the interlayer spaces within the raw bentonite.

The FTIR spectra of the raw bentonite and BB are given in Supplemental Fig. S2. The detected OH stretching of the adsorbed water could be observed within the multiple broad band of 3400–3650 cm^−1^, implied the presence of two types of OH groups related to the hydrogen bonding and isolated OH-group. The shifted from 3622 to 3624 cm^−1^, and 3429 cm^−1^ to 3436 cm^−1^ of adsorption band after the modification could be attributed to the structural OH groups of BB. The aliphatic hydrocarbons of the raw bentonite is ascribed to the sharp peaks at 2925 cm^−1^ and 1431–1433 cm^−1^^53^, while the OH group and Al–Al–OH bending resulting from the dioctahedral montmorillonite 2:1 layer were detected at 1638–1639 cm^−1^ and 916/917 cm^−1^^54^. The strong absorption band at 1035 cm^−1^ and the sharp peak at 796–797 cm^−1^ are due to the Si–O, and the quartz admixture of the clay based-adsorbent, while the intensity at 695–694 cm^−1^ is driven by the deformation of the Si–O bond. The tetrahedral bending modes of Si–O–Al and Si–O–Si could be illustrated at 523 and 466–467 cm^−1^. From the presented analysis, the substitution of the sodium salt of the raw metal ions in the interlayer clay structure, or located into the Si–O sheets hexagonal cavities induced changes in the Si–O vibration modes of the bentonite have led to the reconstruction of the tetrahedral sheets either in the hexagonal holes, or in the previously vacant octahedral sites. The result was supported by Jawad and Abdulhameed^55^, who reported the potential of silanol (≡SiOH), aluminol (≡AlOH, and hydroxyl (–OH) groups as the potential active sites within the mineral edges. Accordingly, electrostatic attraction is considered to be one of the most impactful interaction between the MB dye molecules with the clay based adsorbent. Other governing mechanisms for sorption process are H-bonding between the H atom available on the surface of BB, and the N within atom in the MB dye structure, and n-π between the delocalization of the lone pair electron of O atoms into the π orbital of the dye aromatic rings^56^.

Impenetrability of nitrogen to the interlayer space of clay minerals represents an appropriate method in describing the chemical transformation of the external surface of the functionalized materials. The detailed of porosity structures of the raw bentonite and BB are summarized in Supplemental Table S3. It was evident that a greater porosity development was found at BB, with the higher BET surfaces area of 426.91 m^2^/g, Langmuir surface area of 539.02 m^2^/g, and total pore volume of 0.225 cm^3^/g, respectively, as compared with the raw bentonite. The greater porosity structure could be ascribed to the considerable amount of trapped pores in the range of micro and medium mesopores (20–400 Å) of BB, and this ion valence behaviour is expected to be more important in improving the textural properties of the clay derivatives^57^. The porosity development is interrelated to the stacking level of different elementary layers, to support the formation of outer sphere and adsorption process. The intercalation of the Na^+^ cations during the modification has reduced the number of macropores and large mesopores (> 400 Å), resulting in the dramatic improvement of the textural properties of the raw bentonite. Pore size distribution (PSD) is a model of adsorbent internal structure, elucidating the complex void spaces within the functionalized adsorbent, and the fraction of pore surface of a given shape and size. It is classified into micropores (d < 2 nm), mesopore (d = 2–50 nm) and macropore (d > 50 nm) according to the classification of International Union of Pure and Applied Chemistry (IUPAC) pore dimensions. From the presented pore size given in Supplemental Fig. S3 justified by the Density Functional Theory model, intensive peaks of the pore diameter (40–70 Å) were detected at a vast majority of the mesoporous region, which are capable to support the overall adsorption process. The rising pore volume under NaCl treatment could be driven by the production of finely scattered Si oxides, destruction and leaching of ion mineral, removal of silica compound or X-ray amorphous aluminium, blocking the interlamellar spaces and surface porosity by the voids, cracks or decrease in the mineral particle sizes, in which a larger proportion of the newly formed surfaces may be connected to new pore walls.

## Conclusion

In this work, the sodium salt induced activation of bentonite derived adsorbent at low temperature has been attempted. The specific surface area, and total pore volume was identified to be 426.91 m^2^/g and 0.225 cm^3^/g, respectively. Adopting methylene blue as the model adsorbate, the best chemical impregnation ratio at 1:3 resulted in a monolayer adsorption capacity of 318.38 mg/g, well described by the Langmuir isotherm and pseudo-second order kinetic equations. The adsorption process was feasible, endothermic and spontaneous in nature, supported by different surface active sites, notably silanol, aluminol, and hydroxyl groups. The presented results provide a new insight into the sodium salt assisted activation of high-quality clay-based adsorbents, with a reliable, simple, efficient and economical approach.

## Supplementary Information

Supplementary Information.
