# Supplementary material for: Sodium salt-assisted low temperature activation of bentonite for the adsorptive removal of methylene blue
Source: Sci Rep. 2022 Feb 15;12:2534. doi: 10.1038/s41598-022-06254-z (PMC8847366; doi:10.1038/s41598-022-06254-z)
Supplement: Supplementary file 1 — Supplementary Information. [file 41598_2022_6254_MOESM1_ESM.docx]

**Sodium salt-assisted low temperature activation of bentonite for the adsorptive removal of methylene blue**

**Authors’ name and affiliations:**

Siti Fairos Ab Shattar (S.F.A. Shattar)

River Engineering and Urban Drainage Research Centre (REDAC)

Engineering Campus, Universiti Sains Malaysia

14300 Nibong Tebal, Penang, Malaysia

Email: [ctfairosz89@yahoo.com.my](mailto:ctfairosz89@yahoo.com.my)

**Corresponding author**

Keng Yuen Foo (K.Y. Foo)

River Engineering and Urban Drainage Research Centre (REDAC)

Engineering Campus, Universiti Sains Malaysia

14300 Nibong Tebal, Penang, Malaysia

Email : [k.y.foo@usm.my](mailto:k.y.foo@usm.my) (K. Y. Foo)

Tel: +6045996528; Fax: +6045996926

**Supplemental Fig. 1.** Essential characteristic of the Langmuir isotherm model, separation factor, *R*_L_ as

a function of initial concentration for methhylene blue

4000

3600

3200

2800

2400

2000

1800

1600

1400

1200

1000

800

600

400

0

10

20

30

40

50

**Wavenumber (cm^-1^)**

**Transmittance (%)**

**3622**

**3429**

**1638**

**1431**

**1035**

**917**

**797**

**695**

**523**

**466**

**3624**

**3436**

**2925**

**1639**

**1433**

**916**

**796**

**694**

**523**

**467**

Bentonite

BB

**2925**

**Supplemental Fig. 2.** Fourier-Transform Infrared Spectroscopy of bentonite and BB

# Supplemental Fig. 3. Pore-size distributions of bentonite and BB

**Supplemental Table 1.** Adsorption kinetic parameters for the adsorption of MB onto BB at 30 ºC

| ***C*_0_**  **(mg/L)** | ***q*_e,exp_ (mg/g)** | **Pseudo-first order** | | | | **Pseudo-second order** | | | | **Elovich** | | | | |
| --- | --- | --- | --- | --- | --- | --- | --- | --- | --- | --- | --- | --- | --- | --- |
|  |  | ***q*_e,cal_ (mg/g)** | ***k*_1_ (1/h)** | ***R*^2^** | **Δ*q* (%)** | ***q*_e,cal_ (mg/g)** | ***k*_2_**  **(g/mg h)** | ***R*^2^** | **Δ*q* (%)** | ***q*_e,cal_ (mg/g)** | ***a* (×10^5^)**  **(mg/g h)** | ***b***  **(g/ mg)** | ***R*^2^** | **Δ*q*(%)** |
| 50 | 49.36 | 25.99 | 4.41 | 0.772 | 47.33 | 46.73 | 0.38 | 0.999 | 5.32 | 44.59 | 4.67 | 0.21 | 0.919 | 9.65 |
| 100 | 98.44 | 62.60 | 6.01 | 0.908 | 36.41 | 101.53 | 0.10 | 0.999 | 3.14 | 95.05 | 0.41 | 0.06 | 0.943 | 3.44 |
| 200 | 194.57 | 124.18 | 4.37 | 0.863 | 36.18 | 188.68 | 0.06 | 0.999 | 3.03 | 174.08 | 0.95 | 0.03 | 0.958 | 10.53 |
| 300 | 277.89 | 194.63 | 5.89 | 0.938 | 29.96 | 290.69 | 0.03 | 0.999 | 4.61 | 263.62 | 0.99 | 0.02 | 0.956 | 5.14 |
| 400 | 299.79 | 203.73 | 5.19 | 0.922 | 32.04 | 303.03 | 0.03 | 0.999 | 1.08 | 278.63 | 0.89 | 0.02 | 0.975 | 7.06 |
| 500 | 318.20 | 219.84 | 5.50 | 0.928 | 30.91 | 333.33 | 0.02 | 0.999 | 4.76 | 299.51 | 0.92 | 0.02 | 0.978 | 5.88 |

**Supplemental Table 2.** Intraparticle diffusion model parameters and correlation coefficients for the adsorption of MB onto BB at 30 °C

| ***C*_0_ (mg/L)** | **Intraparticle diffusion model** | | | | | | | | |
| --- | --- | --- | --- | --- | --- | --- | --- | --- | --- |
|  | ***K*_p1_ (mg/g h^1/2^)** | ***K*_p2_ (mg/g h^1/2^)** | ***K*_p3_ (mg/g h^1/2^)** | ***C*_1_** | ***C*_2_** | ***C*_3_** | **(*R*_1_)^2^** | **(*R*_2_)^2^** | **(*R*_3_)^2^** |
| 50 | 67.05 | 9.64 | - | 0 | 34.46 | 49.36 | 0.913 | 0.987 | - |
| 100 | 131.40 | 12.52 | - | 0 | 78.97 | 98.44 | 0.980 | 0.975 | - |
| 200 | 241.13 | 32.89 | 4.54 | 0 | 135.68 | 184.74 | 0.961 | 0.952 | 0.466 |
| 300 | 358.74 | 33.65 | 4.43 | 0 | 223.17 | 268.26 | 0.977 | 0.807 | 0.617 |
| 400 | 374.98 | 43.74 | 10.54 | 0 | 222.50 | 276.35 | 0.975 | 0.916 | 0.879 |
| 500 | 397.97 | 43.88 | 11.04 | 0 | 243.69 | 293.64 | 0.979 | 0.894 | 0.880 |

**Supplemental Table 3.** Surface physical properties of bentonite and BB

| **Properties** | **Bentonite** | **BB** |
| --- | --- | --- |
| BET surface area (m^2^/g) | 120.34 | 426.91 |
| Micropore surface area (m^2^/g) | 11.26 | 64.04 |
| External surface area (m^2^/g) | 109.08 | 362.87 |
| Langmuir surface area (m^2^/g) | 151.75 | 539.02 |
| Total pore volume (cm^3^/g) | 0.155 | 0.225 |
| Micropore volume (cm^3^/g) | 0.017 | 0.034 |
| Mesopore volume (cm^3^/g) | 0.138 | 0.191 |
| Average pore size (Å) | 46.67 | 57.77 |
